# In Vitro and In Vivo Antibacterial Activity of *Punica granatum* Peel Ethanol Extract against *Salmonella*

**DOI:** 10.1093/ecam/nep105

**Published:** 2011-01-12

**Authors:** Jang-Gi Choi, Ok-Hwa Kang, Young-Seob Lee, Hee-Sung Chae, You-Chang Oh, Obiang-Obounou Brice, Min-San Kim, Dong-Hwan Sohn, Hun-Soo Kim, Hyun Park, Dong-Won Shin, Jung-Rae Rho, Dong-Yeul Kwon

**Affiliations:** ^1^College of Pharmacy and Wonkwang-Oriental Medicines Research Institute, Wonkwang University, Iksan, Jeonbuk, 570-749, Republic of Korea; ^2^Department of Pharmacy, College of Pharmacy, Wonkwang University, Republic of Korea; ^3^College of Medicine, Wonkwang University, Jeonbuk 570-749, Republic of Korea; ^4^Department of Oriental Medicine Resources, Sunchon National University, Jeonnam 540-742, Republic of Korea; ^5^Department of Oceanography, Kunsan National University, Jeonbuk 573-701, Republic of Korea

## Abstract

*Punica granatum* is commonly used in Korea as a traditional medicine for the treatment of pathogenic bacteria. In this study, we investigated the *in vitro* and *in vivo* antimicrobial activity of *P. granatum* peel EtOH extract (PGPE) against 16 strains of *Salmonella*. The minimal inhibitory concentrations of PGPE were in the range of 62.5–1000 x03BCg mL^−1^. In addition, the *in vivo* antibacterial activity of the PGPE extract was examined in a *S. typhimurium* infection mouse model. Mice were initially infected with *S. typhimurium* and then with PGPE. The extract was found to have significant effects on mortality and the numbers of viable *S. typhimurium* recovered from feces. Although clinical signs and histological damage were rarely observed in the treated mice, the untreated controls showed signs of lethargy and histological damage in the liver and spleen. Taken together, the results of this study indicate that PGPE has the potential to provide an effective treatment for salmonellosis.

## 1. Introduction

*Salmonella enterica*, which are Gram-negative bacterial pathogens capable of infecting humans and animals, cause significant morbidity and mortality worldwide [1]. *S. enterica* serovar *typhimurium* is a clinically important intracellular bacterial pathogen that causes food poisoning and gastroenteritis in millions of people worldwide each year [2]. The Centers for Disease Control (CDC) estimates that there are nearly 1.4 million food-borne *Salmonella* infections annually in the USA [3]. This bacterium infects the intestinal tract and causes systemic infection of various organs such as the liver and spleen [4].

Fluoroquinolones and tetracyclines are the antibiotics most commonly used to treat *Salmonella*, and until recently most strains were susceptible to these drugs. However, a high incidence of *Salmonella* strains resistant to commonly prescribed antibiotics has recently been reported in Korea and other countries [5, 6], and the increased appearance of antibiotic resistant strains of *Salmonella* further exacerbates this problem [7]. One major concern to public health has been the global dissemination of *S. typhimurium* Definitive Type 104, which is commonly resistant to five or more antimicrobial agents [8–11]. The rise in antibiotic-resistant pathogens has led to the development of new therapeutic agents that are effective against these bacteria. Recently, there has been considerable interest in the use of plant materials as an alternative method to control pathogenic microorganisms [12], and many compounds of plant products have been shown to be specifically targeted against resistant pathogenic bacteria [13].

*Punica granatum*, which belongs to the family of Punicaceae, is commonly known as pomegranate, grenade, granats and punica apple [14]. *Punica granatum* has been used extensively as a traditional medicine in many countries [15] for the treatment of dysentery, diarrhea, helminthiasis, acidosis, hemorrhage and respiratory pathologies [16, 17]. In addition, *P. granatum* is reported to have antioxidant [18, 19], anti-atherosclerotic [20, 21], antibacterial [22, 23] and antiviral [24] properties. The constituents of *P. granatum* include gallocatechins, delphinidin, cyanidin, gallic acid, ellagic acid, pelargonidin and sitosterol, which are very well known for their therapeutic properties [25].

*Punica granatum* peel is used to treat infections found in human sexual organs as well as mastitis, acne, folliculitis, pile, allergic dermatitis, tympanitis, scalds, diarrhea, dysentery and as an antioxidant [26]. In addition, it is reported that the extracts of *P. granatum* have antimicrobial activity against *Salmonella* [23]. However, to date, no studies regarding the antimicrobial activity of *P. granatum* peels have been conducted. Therefore, the goal of this study is to evaluate the antimicrobial activity of the EtOH extract of *P. granatum* peel using various *in vitro* and *in vivo* models.

## 2. Methods

### 2.1. Bacterial Strains and Culture Medium

*Salmonella typhi* (ATCC 19943), *S. dublin* (ATCC 39184), *S. derby* (ATCC 6960), *S. choleraesuis* (ATCC 7001) and *S. gallinarum* (ATCC 9184) were used in this study (Table 1). In addition, this study included local isolates of *S. enteritidis, S. typhimurium, S. gallinarum* and *S. paratyphi* A, which were provided by the National Veterinary Research and Quarantine Service, Republic of Korea. Bacterial strains were suspended in Mueller Hinton broth (MHB, Difco, USA) and then incubated at 37°C for 20 h. Mueller Hinton agar (MHA, Difco) was used for the agar diffusion method and minimal inhibitory concentration (MIC). *Salmonella typhimurium* (JOL 389) was used for *in vivo* assays in mice.

### 2.2. Extraction of Plant Material

*Punica granatum* peel was purchased from an Oriental drug store, Daehak Hanyakkuk (Iksan, Korea), and then authenticated by Dr D.Y. Kwon. A voucher specimen (no. 06-022) was deposited in the Laboratory of Herbalogy, College of Pharmacy, Wonkwang University, Iksan, Korea. Next, the *P. granatum* peel was air-dried in the dark at room temperature and then ground into a powder using a mechanical grinder. Approximately 500 g of the powdered materials were then boiled in 1500 mLof EtOH for 3 h. The solvent was then removed under reduced pressure in a rotary evaporator (N-1000S, EYELA, Japan) and dissolved in water or 50% dimethyl sulfoxide (DMSO, Sigma, USA) prior to use.

### 2.3. High-Performance Liquid Chromatography Analysis

**Figure 1:**
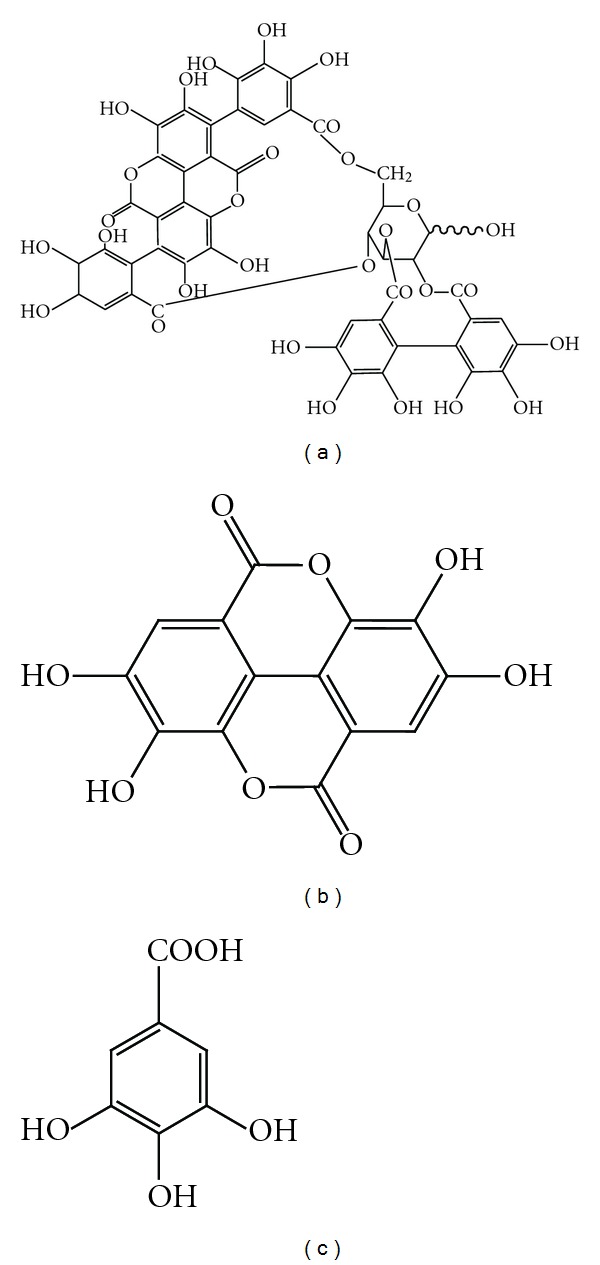
The chemical structure of punicalagin (a), ellagic acid (b) and gallic acid (c).

**Figure 2:**
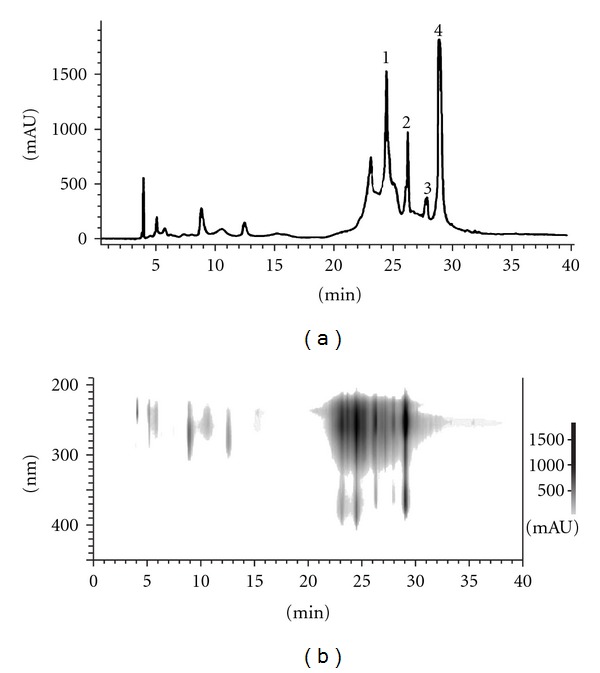
HPLC analysis of PGPE (a) and 3D HPLC analysis (b). (1) and (2) punicalagin isomers; (3) gallic acid; (4) ellagic acid.

The High-performance liquid chromatography (HPLC) system consisted of a Shimadzu LC-6A model (Shimadzu, Tokyo, Japan), with a column of ODS-C18 (4.6 × 250 mm, 5 *μ*m) and a detection of SPD- 6AV with a sensitivity of 0.04 AUFS and a wavelength of 254 nm. Elution was carried out at a flow rate of 0.8 mL/min under a linear gradient of acetonitrile (solvent A) and H_2_O with 1% formic acid (solvent B) from 5% A to 100% A in 50 min. The *P. granatum* peel EtOH extract (PGPE) was dissolved in a mixture of methanol and water (6 : 4 v v^−1^), and 20 *μ*lL was injected into the HPLC. The presence of gallic acid and ellagic acid was confirmed by the same retention time of their standards (Sigma Chemical Co, St Louis, USA) (Figure 1). The obtained chromatogram is shown in Figure 2 [27].

### 2.4. Antimicrobial Resistance Testing

The resistance of the various *Salmonella* strains to different antimicrobial agents was determined using the disk-agar method standardized by the Clinical and Laboratory Standards Institute [28]. The quality control strain used was *Enterococcus faecalis* ATCC 29212.

### 2.5. Disc Diffusion Method

The antibacterial activities of the isolates on the different extracts were tested using the disk-agar method described by the Clinical and Laboratory Standards Institute Standards and by using a modified agar-well diffusion method [28, 29]. Briefly, sterile paper discs (6 mm; Toyo Roshi Kaihsa, Japan) were loaded with 20 *μ*L of PGPE (varying concentrations: 100, 200 and 500 *μ*g) dissolved in 50% DMSO and then left to dry for 18 h at 37°C in a sterile room. The bacterial suspensions were then diluted to a turbidity of approximately 0.5 McFarland (*∼*1.5 × 10^8^ CFU mL^−1^), and then further diluted to obtain the final inoculum. Next, the MHA was poured into Petri dishes and inoculated with 100 *μ*lL of the suspension containing 1 × 10^5^ CFU mL^−1^ of bacteria. Ampicillin (Sigma Chemical Co) was used as the positive control and discs treated with 50% DMSO were used as the negative control. The plates were then placed in an incubator (Vision Co, Seoul, Korea) at 37°C for 24 h, after which the diameter of the zone of inhibition around each of the discs was measured and recorded. Each experiment was performed in triplicate.

### 2.6. Determination of MICs

The MIC values were determined for microorganisms that were found to be sensitive to PGPE during the disc diffusion assay. To accomplish this, the microorganism inocula were prepared from 12-h broth cultures and the suspensions were then adjusted to a turbidity of 0.5 McFarland. Susceptibility tests were then conducted using the standard broth micro dilution method in accordance with the CLSI guidelines [30] in MHB with an inoculum of *∼*5 × 10^4^ CFU mL^−1^. The MHB was then supplemented with serial dilutions of *P. granatum* peel of EtOH extracts ranging from 3.9 to 2000 *μ*g mL^−1^ and ampicillin concentration was ranging from 0.03 to 250 *μ*g mL^−1^. The lowest concentration of PGPE capable of inhibiting visible growth after 24 h of incubation at 37°C was then recorded as the MIC [30].

### 2.7. Animals

Mice were obtained from Da Mool Science (Deajeon, Korea). All mice experiments in this study were approved by the Wonkwang University Animal Ethics Committee in accordance with the guidelines of the Korean Council on Animal Care. Fifteen male Balb/c mice (15–17 g) aged between 5 and 6 weeks were used for all *in vivo* experiments. They were kept in a temperature-controlled room under a 12 h light 12 h dark cycle. Animals had free access to commercial solid food (SCF Co. Ltd, Korea) and water *ad libitum*, and were acclimatized for at least 1 week prior to beginning the experiments.

### 2.8. In Vivo Assay Using Mice

Mice were divided into the following groups: control (CON), *Salmonella*-infected (SI) and *Salmonella*-infected + PGPE (SIPG). Each treatment group contained five mice. Throughout the experiment, mice were provided with water that contained streptomycin (5 mg mL^−1^) in order to reduce the level of facultative anaerobic bacteria that normally colonize the mouse intestine [31]. The inhibition of the growth of test organisms in mice was then determined by monitoring *S. typhimurium* in the feces of the mice. Briefly, *S. typhimurium* (JOL 389) was grown overnight in Luria–Bertani broth (Difco), centrifuged, washed in phosphate-buffered saline (PBS) and then diluted into 20% sucrose to achieve a final concentration of 1 × 10^5^ CFU. The SI and SIPG groups exclusively were then inoculated using gavage needle orally with approximately 10^5^ CFU of *S. typhimurium* in a 0.1 mL volume. One hour after infection, animals in the SIPG group were orally administered 5 mg (using gavage needle) of the PGPE daily, whereas CON and SI animals were not. Fecal samples were then collected 0, 1, 2, 3, 4, 5 and 6 days after the bacterial suspensions were administered and the numbers of the bacteria per gram of feces were determined. Aliquots (100 *μ*l) of fecal suspensions were serially diluted in PBS and then plated on duplicate *Salmonella*-*Shigella* agar plates (Difco), which were subsequently incubated overnight at 37°C. Typical colonies were then counted on plates that contained between 30 and 300 colonies [32], after which confirmation of *S. typhimurium* was performed by a PCR assay using a previously described method [33]. At Day 4 post-infection, the mice were sacrificed, and tissue specimens of the kidney, liver, intestine and spleen organs were transferred to 10% buffered neutral formalin for histopathologic examinations and then processed using standard procedures. Sections of paraffin-embedded tissues were then stained with hematoxylin and eosin.

## 3. Results

### 3.1. Determination of Antibacterial Activity by the Disc Diffusion Method

The antimicrobial efficacy of PGPE against the 16 *Salmonella* strains was evaluated by the disc diffusion method via determination of the surrounding zones of inhibition, as well as by evaluating the MIC using the agar dilution method.  Table 2 shows the antimicrobial activity of *P. granatum* peel extract as determined by the disc diffusion method. The mean values of the zones of inhibition produced against the tested bacteria ranged from 13.3 to 18.6 mm, with the growth of each of the tested strains being inhibited at 500 *μ*g per disc and the zone of inhibition increasing in a dose-dependant manner.

### 3.2. Determination of MICs

The MICs of the PGPE against the 16 strains of *Salmonella* are shown in Table 3. The MICs determined using the broth dilution method confirmed the results obtained using the disc diffusion method. PGPE showed antimicrobial activity against each of the tested strains, and these values ranged from 62.5 to 1000 *μ*g mL^−1^. The *in vivo* experiment was therefore conducted with the EtOH extract.

### 3.3. Antibacterial Efficacy of PGPE in Mice

**Figure 3:**
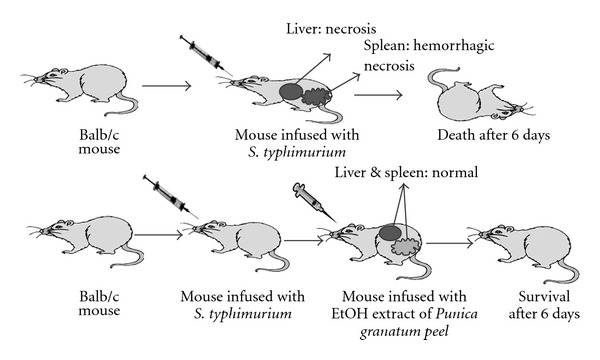
Importance of PGPE against *S. typhimurium* infection.

The *in vivo* antibacterial activity of PGPE was examined using a mouse *S. typhimurium* infection model. Briefly, mice were infected with 1 × 10^5^ CFU of *S. typhimurium* (SI). One-hour later, the mice were orally administered PGPE (SIPG). As shown in Table 4, treatment with the extract of *P. granatum* peel was found to have marked effects on mortality and on the number of viable *S. typhimurium* recovered from feces. At Day 1 post-infection, ten mice in the SI and SIPG group shed viable *S. typhimurium* in feces, with the feces of mice in the SI group being found to contain bacteria at a concentration of 3 × 10^3^ to 4 × 10^5^ CFU g^−1^ and feces of mice in the SIPG group being found to contain bacteria at a concentration of 2 × 10^2^ to 2 × 10^3^ CFU g^−1^. In addition, at Day 6 post-injection, none of the mice in the SIPG group had died, whereas all five mice in the SI group had succumbed as illustrated in Figure 3.

### 3.4. Organ Histopathologic Changes

**Figure 4:**
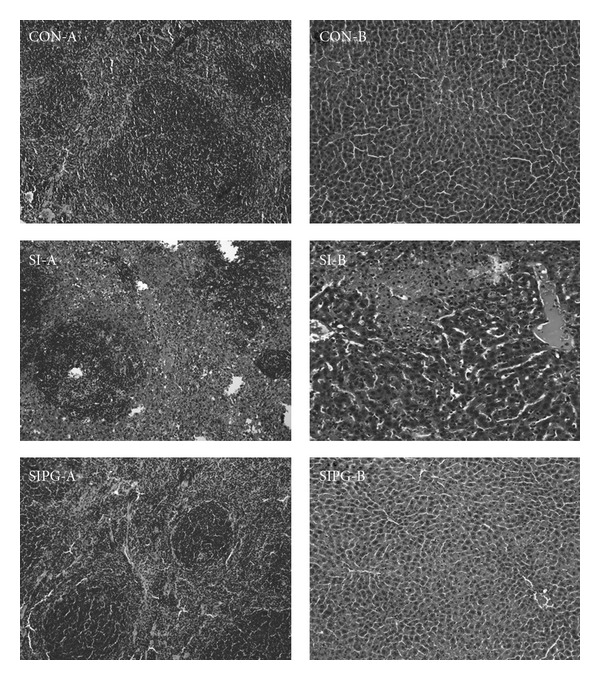
Histopathological changes in organs in CON, SI and SIPG. (a) spleen (×200) and (b) liver (×200).

*Salmonella typhimurium*-infected mice that did not receive the PGPE were lethargic and showed signs of histological damage in the liver and spleen. In addition, the central and portal veins of the liver showed congestion with focal necrotic emboli-like materials (Figure 4). There were multiple small necrotizing nodular lesions in the liver parenchyma with Kuffer cell hyperplasia and inflammatory cell infiltrate. The spleen showed extensive hemorrhagic necrosis in the red pulp with multiple apoptotic bodies in the white pulp (Figure 4). No specific abnormal findings were observed in the kidney or the small intestine. Conversely, clinical signs and histological damage were rarely observed in *S. typhimurium*-infected mice fed with the PGPE.

## 4. Discussion

Recently, a number of antibiotics have lost their effectiveness due to the development of resistant strains of bacteria, which has primarily occurred through the expression of resistance genes [34, 35]. In addition to inducing resistance, antibiotics are sometimes associated with opposing effects such as hypersensitivity, immune-suppression and allergic reactions [36]. Therefore, there is a need to develop alternative antimicrobial drugs for the treatment of infectious diseases [37, 38].

In the present study, the PGPE exhibited antibacterial activity against all 16 strains of eight different *Salmonella* serotypes tested. In addition, the results of the MIC assays also confirmed its antibacterial effects against all tested *salmonella* strains. The PGPE also exhibited antibacterial activities against *Salmonella* strains JOL 389, JOL 411, JOL 419, JOL 420, JOL 421 and JOL 423, all of which have been shown to be resistant to two to five antibiotics (Table 1). The *in vivo* antibacterial assay also revealed that the extract effectively inhibited the growth of *S. typhimurium* and significantly reduced mouse mortality (Table 4). Furthermore, clinical signs of infection and histological damage were rarely observed in test mice, whereas untreated SI mice showed severe clinical signs and histological damage in the tested organs. This is the first study describing the antibacterial activity of *P. granatum* peel extract against *Salmonella*. Based on these promising *in vitro* and *in vivo* assay findings, we believe that *P. granatum* peel extract is likely to become a novel antimicrobial treatment for salmonellosis. It has been reported that *P. granatum* peel extracts have shown antibacterial activity against *Escherichia coli* O157 and methicillin-resistant *Staphylococcus aureus* bacteria [14, 39]. This antibacterial activity may be indicative of the presence of several metabolic toxins or broad-spectrum antibiotics. Several metabolites from herb species, including alkaloids, tannins and sterols, have previously been associated with antimicrobial activity [40].

In order to investigate components from the PGPE, the HPLC analysis was performed as shown in Figures 2(a) and 2(b). This HPLC analysis among some other minor constituents mainly shows some major phenolic compounds [26]; gallic acid and ellagic acids in addition to punicalagin as a major ellagitannin. The retention time shows it to be gallic acid and ellagic acid [41, 42] and also the presence of punicalagin isomers (Figure 1) could be deduced to be one of the major components from the results of literature reported previously [27]. Gallic acid was reported to have antibacterial activity against some intestinal bacteria [43], ellagic acid has anti-microbial activity [44] and punicalagin was reported to show anti-food-borne pathogens [45]. The site and the number of hydroxyl groups on the phenol components may increase the toxicity against the microorganisms [46]. However, it has been reported that gallic acid, ellagic acid and punicalagin have weak antibacterial activity against *Salmonella*.

The antibacterial activity of *P. granatum* peel extract might be related to the action of its antibiotic compounds or to the presence of metabolic toxins. This suggests that these components may also provide antibacterial activity against *Salmonella* and provide a plausible explanation for the higher antibacterial activity of the EtOH extract. On the other hand, the unknown minor components present have not been elucidated in terms of their activity. Further studies then need to be done. In the future, thorough investigation is needed to better ascertain the antibacterial effect of this herb extract.

## Funding

Grant No. RTI 05-03-02 from the Regional Technology Innovation Program of the Ministry of Commerce, Industry and Energy (MOCIE) in Republic of Korea.

## Figures and Tables

**Table 1:** List of *Salmonella* strains used in this study.

Strain	Serotypes	Origin	Resistant antibiotics
JOL 380	*S. typhi* ATCC 19943	Human	—
JOL 381	*S. paratyphi* A	Human	—
JOL 386	*S. enteritidis*	Chicken	—
JOL 387	*S. typhimurium*	Cattle	—
JOL 388	*S. typhimurium*	Cattle	—
JOL 389	*S. typhimurium*	Pig	AM, C, G, S, TIC
JOL 407	*S. enteritidis*	Chicken	—
JOL 408	*S. typhimurium*	Pig	—
JOL 409	*S. dublin* ATCC 39184	Cattle	—
JOL 410	*S. derby* ATCC 6960	Pig	—
JOL 411	*S. choleraesuis* ATCC 7001	Pig	AM, SXT
JOL 419	*S. gallinarum*	Chicken	CF, G, SXT
JOL 420	*S. gallinarum*	Chicken	CF, CIP, NA
JOL 421	*S. gallinarum*	Chicken	G, NA, S
JOL 422	*S*. *gallinarum*	Chicken	—
JOL 423	*S. gallinarum* ATCC 9184	Chicken	AM, AMC, C, G, S

AM, ampicillin; AMC, amoxicillin/clavulanic acid; C, chloramphenicol; CF, cephalothin; G, sulfisoxazole; NA, nalidixic acid; S, streptomycin; SXT, trimethoprim/sulfamethoxazole; TIC, ticarcillin.

**Table 2:** Antimicrobial activity (as inhibition zone diameters) of PGPE and ampicillin (APCL) against 16 strains of *Salmonella*.

Strain	Serotypes	Diameter of clear zone (mm)			
		PGPE			APCL^a^
		100 *μ*g	200 *μ*g	500 *μ*g	10 *μ*g
JOL 380	*S. typhi* ATCC 19943	13.3 ± 1.1	16.3 ± 1.5	17.3 ± 1.1	34.2 ± 1.0
JOL 381	*S. paratyphi* A	14.3 ± 0.5	15.6 ± 1.1	18.6 ± 1.1	28.5 ± 1.1
JOL 386	*S. enteritidis*	9.0 ± 1.0	12.6 ± 1.5	14.3 ± 1.1	31.1 ± 0.3
JOL 387	*S. typhimurium*	9.6 ± 0.5	11.6 ± 0.5	14.6 ± 0.5	27.5 ± 1.0
JOL 388	*S. typhimurium*	11.0 ± 1.0	14.0 ± 1.0	15.0 ± 1.0	26.0 ± 1.0
JOL 389	*S. typhimurium*	9.0 ± 1.0	12.0 ± 1.0	12.6 ± 0.5	ND
JOL 407	*S. enteritidis*	9.3 ± 1.1	12.3 ± 0.5	14.6 ± 0.5	26.7 ± 1.2
JOL 408	*S. typhimurium*	8.6 ± 1.1	10.6 ± 0.5	14.0 ± 2.0	30.2 ± 0.5
JOL 409	*S. dublin* ATCC 39184	8.0 ± 0.0	10.0 ± 0.0	13.3 ± 0.5	27.0 ± 1.1
JOL 410	*S. derby* ATCC 6960	10.0 ± 0.0	12.0 ± 0.0	14.3 ± 0.5	25.6 ± 1.1
JOL 411	*S. choleraesuis* ATCC 7001	11.6 ± 0.5	15.6 ± 1.0	16.0 ± 0.0	11.2 ± 1.0
JOL 419	*S. gallinarum*	12.3 ± 0.5	16.0 ± 1.0	16.6 ± 1.1	27.3 ± 1.5
JOL 420	*S. gallinarum*	16.0 ± 0.0	16.0 ± 0.7	17.6 ± 0.5	25.8 ± 1.1
JOL 421	*S. gallinarum*	11.3 ± 0.5	15.0 ± 0.0	16.3 ± 0.5	25.2 ± 0.3
JOL 422	*S*. *gallinarum*	12.0 ± 0.0	14.0 ± 1.7	16.0 ± 1.0	28.5 ± 0.5
JOL 423	*S. gallinarum* ATCC 9184	7.6 ± 0.5	10.0 ± 0.0	13.3 ± 1.1	ND

Data shown represent the mean ± SE of three experiments that consisted of three replicates. ND, No activity detected.

^a^Positive control.

**Table 3:** Antimicrobial activity of PGPE and ampicillin (APCL) against 16 strains of *Salmonella*.

Strain	Serotypes	MIC (*μ*g mL^−1^)	
		PGPE	APCL^a^
JOL 380	*S. typhi* ATCC 19943	250	0.97
JOL 381	*S. paratyphi* A	62.5	1.95
JOL 386	*S. enteritidis*	1000	1.95
JOL 387	*S. typhimurium*	1000	1.95
JOL 388	*S. typhimurium*	500	0.97
JOL 389	*S. typhimurium*	250	*>*250
JOL 407	*S. enteritidis*	250	1.95
JOL 408	*S. typhimurium*	500	1.95
JOL 409	*S. dublin* ATCC 39184	500	0.97
JOL 410	*S. derby* ATCC 6960	500	1.95
JOL 411	*S. choleraesuis* ATCC 7001	62.5	*>*250
JOL 419	*S. gallinarum*	62.5	1.95
JOL 420	*S. gallinarum*	62.5	1.95
JOL 421	*S. gallinarum*	125	1.95
JOL 422	*S*. *gallinarum*	250	1.95
JOL 423	*S. gallinarum* ATCC 9184	1000	>250

^a^Positive control.

**Table 4:** Effects of treatment with PGPE on fecal shedding of *S. typhimurium* (CFU g^−1^) by mice.

Group	Day of post-feeding						
	Day 0	Day 1	Day 2	Day 3	Day 4	Day 5	Day 6
SI-1	0	3 × 10^3^	2 × 10^3^	1 × 10^4^	2 × 10^4^	1 × 10^4^	Death
SI-2	0	2 × 10^4^	5 × 10^4^	7 × 10^5^	Death	Death	Death
SI-3	0	3 × 10^4^	1 × 10^5^	2 × 10^5^	Death	Death	Death
SI-4	0	7 × 10^4^	3 × 10^4^	2 × 10^4^	1 × 10^6^	Death	Death
SI-5	0	4 × 10^5^	2 × 10^5^	1 × 10^5^	3 × 10^6^	Death	Death
SIPG-1	0	1 × 10^3^	1 × 10^3^	1 × 10^3^	6 × 10^3^	2 × 10^3^	0
SIPG-2	0	2 × 10^3^	1 × 10^3^	2 × 10^3^	3 × 10^3^	2 × 10^3^	2 × 10^3^
SIPG-3	0	2 × 10^2^	2 × 10^3^	8 × 10^3^	3 × 10^3^	3 × 10^2^	2 × 10^2^
SIPG-4	0	1 × 10^3^	3 × 10^2^	2 × 10^2^	1 × 10^2^	3 × 10^2^	2 × 10^2^
SIPG-5	0	4 × 10^2^	8 × 10^2^	1 × 10^3^	2 × 10^2^	3 × 10^2^	1 × 10^2^

## References

[1] Fink SL, Cookson BT (2007). Pyroptosis and host cell death responses during *Salmonella* infection. *Cellular Microbiology*.

[2] Grassl GA, Valdez Y, Bergstrom KSB, Vallance BA, Finlay BB (2008). Chronic enteric *Salmonella* infection in mice leads to severe and persistent intestinal fibrosis. *Gastroenterology*.

[3] Mead PS, Slutsker L, Dietz V (1999). Food-related illness and death in the United States. *Emerging Infectious Diseases*.

[4] Coburn B, Grassl GA, Finlay BB (2007). *Salmonella*, the host and disease: a brief review. *Immunology and Cell Biology*.

[5] Choi S-H, Woo JH, Lee JE (2005). Increasing incidence of quinolone resistance in human non-typhoid *Salmonella* enterica isolates in Korea and mechanisms involved in quinolone resistance. *Journal of Antimicrobial Chemotherapy*.

[6] Stevenson JE, Gay K, Barrett TJ, Medalla F, Chiller TM, Angulo FJ (2007). Increase in nalidixic acid resistance among non-typhi *Salmonella* enterica isolates in the United States from 1996 to 2003. *Antimicrobial Agents and Chemotherapy*.

[7] Bhan MK, Bahl R, Bhatnagar S (2005). Typhoid and paratyphoid fever. *The Lancet*.

[8] Gebreyes WA, Thakur S, Davies PR, Funk JA, Altier C (2004). Trends in antimicrobial resistance, phage types and integrons among *Salmonella* serotypes from pigs, 1997–2000. *Journal of Antimicrobial Chemotherapy*.

[9] Perron GG, Bell G, Quessy S (2008). Parallel evolution of multidrug-resistance in *Salmonella* enterica isolated from swine. *FEMS Microbiology Letters*.

[10] Poppe C, Ziebell K, Martin L, Allen K (2002). Diversity in antimicrobial resistance and other characteristics among *Salmonella typhimurium* DT104 isolates. *Microbial Drug Resistance*.

[11] Threlfall EJ, Ward LR, Frost JA, Willshaw GA (2000). Spread of resistance from food animals to man–the UK experience. *Acta Veterinaria Scandinavica. Supplementum*.

[12] Aqil F, Khan MS, Owais M, Ahmad I (2005). Effect of certain bioactive plant extracts on clinical isolates of beta-lactamase producing methicillin resistant *Staphylococcus aureus*. *Journal of Basic Microbiology*.

[13] Nostro A, Cellini L, Di Bartolomeo S (2006). Effects of combining extracts (from propolis or *Zingiber officinale*) with clarithromycin on *Helicobacter pylori*. *Phytotherapy Research*.

[14] Voravuthikunchai SP, Sririrak T, Limsuwan S, Supawita T, Iida T, Honda T (2005). Inhibitory effects of active compounds from *Punica granatum* pericarp on verocytotoxin production by enterohemorrhagic *Escherichia coli* O157:H7. *Journal of Health Science*.

[15] Singh RP, Chidambara MKN, Jayaprakasha GK (2002). Studies on the antioxidant activity of pomegranate (*Punica granatum*) peel and seed extracts using in vitro models. *Journal of Agricultural and Food Chemistry*.

[16] Ricci D, Giamperi L, Bucchini A, Fraternale D (2006). Antioxidant activity of *Punica granatum* fruits. *Fitoterapia*.

[17] Sánchez-Lamar A, Fonseca G, Fuentes JL (2007). Assessment of the genotoxic risk of *Punica granatum* L. (Punicaceae) whole fruit extracts. *Journal of Ethnopharmacology*.

[18] Related A, LinksHeber D, Seeram NP (2007). Safety and antioxidant activity of a pomegranate ellagitannin-enriched polyphenol dietary supplement in overweight individuals with increased waist size. *Journal of Agricultural and Food Chemistry*.

[19] Parmar HS, Kar A (2008). Medicinal values of fruit peels from *Citrus sinensis*, *Punica granatum*, and Musa paradisiaca with respect to alterations in tissue lipid peroxidation and serum concentration of glucose, insulin, and thyroid hormones. *Journal of Medicinal Food*.

[20] Aviram M, Rosenblat M, Gaitini D (2004). Pomegranate juice consumption for 3 years by patients with carotid artery stenosis reduces common carotid intima-media thickness, blood pressure and LDL oxidation. *Clinical Nutrition*.

[21] Parmar HS, Kar A (2007). Protective role of Citrus sinensis, Musa paradisiaca, and *Punica granatum* peels against diet-induced atherosclerosis and thyroid dysfunctions in rats. *Nutrition Research*.

[22] Braga LC, Shupp JW, Cummings C (2005). Pomegranate extract inhibits *Staphylococcus aureus* growth and subsequent enterotoxin production. *Journal of Ethnopharmacology*.

[23] Naz S, Siddiqi R, Ahmad S, Rasool SA, Sayeed SA (2007). Antibacterial activity directed isolation of compounds from *Punica granatum*. *Journal of Food Science*.

[24] Zhang J, Zhan B, Yao X, Gao Y, Shong J (1995). Antiviral activity of tannin from the pericarp of *Punica granatum* L. against genital Herpes virus in vitro. *Zhongguo Zhong yao za zhi*.

[25] Lansky EP, Newman RA (2007). *Punica granatum* (pomegranate) and its potential for prevention and treatment of inflammation and cancer. *Journal of Ethnopharmacology*.

[26] Singh RP, Chidambara MKN, Jayaprakasha GK (2002). Studies on the antioxidant activity of pomegranate (*Punica granatum*) peel and seed extracts using in vitro models. *Journal of Agricultural and Food Chemistry*.

[27] Kwak HM, Jeong HH, Sohng BH (2005). Quantitative analysis of antioxdants in Korea pomegranate Husk (Granati pericarpium) cultivated in different site. *Journal of the Korean Society for Applied Biological Chemistry*.

[29] Okunji CO, Okeke CN, Gugnani HC, Iwu MM (1990). An antifungal spirostanol saponin from fruit pulp of *Dracaena mannii*. *International Journal of Crude Drug Research*.

[31] Myhal ML, Laux DC, Cohen PS (1982). Relative colonizing abilities of human fecal and K-12 stains of *Escherichia coli* in the large intestines of streptomycin treated mice. *European Journal of Clinical Microbiology*.

[32] Lee M-H, Kwon HA, Kwon D-Y (2006). Antibacterial activity of medicinal herb extracts against *Salmonella*. *International Journal of Food Microbiology*.

[33] Alvarez J, Sota M, Vivanco AB (2004). Development of a multiplex PCR technique for detection and epidemiological typing of *Salmonella* in human clinical samples. *Journal of Clinical Microbiology*.

[34] Davies J (1994). Inactivation of antibiotics and the dissemination of resistance genes. *Science*.

[35] Service RF (1995). Antibiotics that resist resistance. *Science*.

[36] Ahmad I, Mehmood Z, Mohammad F (1998). Screening of some Indian medicinal plants for their antimicrobial properties. *Journal of Ethnopharmacology*.

[37] Berahou A, Auhmani A, Fdil N, Benharref A, Jana M, Gadhi CA (2007). Antibacterial activity of *Quercus ilex* bark’s extracts. *Journal of Ethnopharmacology*.

[38] Salomão K, Pereira PRS, Campos LC (2008). Brazilian propolis: correlation between chemical composition and antimicrobial activity. *Evidence-Based Complementary and Alternative Medicine*.

[39] Machado TDB, Leal ICR, Amaral ACF, Dos Santos KRN, Da Silva MG, Kuster RM (2002). Antimicrobial ellagitannin of *Punica granatum* fruits. *Journal of the Brazilian Chemical Society*.

[40] Leven MD, Vanden BDA, Marten T, Vilientmick A, Lomweas EC (1979). Screening of higher plants for biological activity. *Planta Medica*.

[41] Lu J, Wei Y, Yuan Q (2007). Preparative separation of punicalagin from pomegranate husk by high-speed countercurrent chromatography. *Journal of Chromatography B*.

[42] Aviram M, Volkova N, Coleman R (2008). Pomegranate phenolics from the peels, arils, and flowers are antiatherogenic: studies in vivo in atherosclerotic apolipoprotein e-deficient (E0) mice and in vitro in cultured macrophages and lipoproteins. *Journal of Agricultural and Food Chemistry*.

[43] Ahn Y-J, Lee C-O, Kweon J-H, Ahn J-W, Park J-H (1998). Growth-inhibitory effects of Galla Rhois-derived tannins on intestinal bacteria. *Journal of Applied Microbiology*.

[44] Thiem B, Goślińska O (2004). Antimicrobial activity of *Rubus chamaemorus* leaves. *Fitoterapia*.

[45] Taguri T, Tanaka T, Kouno I (2004). Antimicrobial activity of 10 different plant polyphenols against bacteria causing food-borne disease. *Biological and Pharmaceutical Bulletin*.

[46] Cowan MM (1999). Plant products as antimicrobial agents. *Clinical Microbiology Reviews*.

